# Gluten Rhapsody

**DOI:** 10.3390/nu11030589

**Published:** 2019-03-11

**Authors:** Luca Elli, Beatrice Marinoni

**Affiliations:** Center for the Prevention and Diagnosis of Coeliac Disease, Division of Gastroenterology and Endoscopy, Fondazione IRCCS Ca’ Granda Ospedale Maggiore Policlinico, Milan 20122, Italy; beatrice.marinoni@unimi.it

**Keywords:** gluten, gluten-free diet, coeliac disease, non-celiac gluten sensitivity, non-coeliac wheat sensitivity, gliadin, microbiota

For decades, gluten-free dieting (GFD) has been accepted as the only therapeutic approach to coeliac disease (CD) and, more recently, for non-coeliac gluten sensitivity (NCGS), a term to refer to the so-called gluten-related disorders (GRD) [1]. 

GFD has become popular among the general population for its supposed beneficial effects on human health [2]. GFD is also the most frequently suggested dietary regimen in irritable bowel syndrome (IBS) [3].

In fact, there are several concerns and misconceptions regarding GFD, which deserve special attention. For such a reason, this Special Issue on “Gluten-Free Diet” comprises 23 peer-reviewed papers, reporting on the most recent evidence and topics about GFD. In particular, the impact of GFD on human health and quality of life; the emerging evidence of its beneficial effects in IBS; and the difficult problems of compliance, costs, and availability of GF food are discussed. 

Several sources of evidence support the notion that, despite its remarkable effectiveness in remitting the vast majority of GRD symptoms, GFD comes with both a social and financial burden. Gluten-free foods are still less available and more expensive than their gluten-containing versions [4], thus causing patients social and psychological consequences in securing good quality of life and compliance with the advised dietary regimen.

As Joelson AM et al. have shown, the prevalence of depression among the sufferers of coeliac disease (CD) is high and depressive symptoms may mask the relationship between symptoms and inadvertent gluten exposure, and thus make it more difficult to detect any lack of adherence to GFD [5]. With a systematic review and meta-analysis, Busby at al. have confirmed that gluten elimination may well represent an effective treatment strategy for mood disorders for individuals with GRD [6].

As permanent adherence to GFD is difficult, with repeated transgression and frequent contaminations, a reliable tool to assess compliance is currently advocated [7]. In their review, Rodrigo L. et al. establish that the determination of the immunogenic gluten peptides in isolated samples of faeces or urine has proven useful for assessing adherence to GFD [8].

In the opinion of some authors, several factors contribute to greater adherence to GFD; that is, good knowledge of the disease and its treatment, high level of education, high economic status, female sex, young age, high self-esteem, good grades at school, good availability and labelling of products, good contact with a doctor and a dietitian, and finally membership of the Coeliac Society [9,10,11,12]. Conversely, the factors responsible for not adhering to GFD are poor taste of gluten-free products, high price and poor availability, being adolescent, the absence of immediate symptoms following the intake of small amounts of gluten, and low awareness of the disease [9,11,12]. 

From a study conducted by Czaja-Bulsa et al., it has emerged that GFD adherence has improved over the last ten years, thanks to the popularity gained by GFD and GF food becoming more available [13].

Further evidence, recently accepted, shows that in spite of improvements in food formulation over the last few years, GF foods still present with a reduced nutritional profile when compared with gluten-containing products, with higher lipid and trans-fat content; lower level of proteins; and lower degree of fortification with micronutrients, especially Ca, Fe, Mg, and Zn [14]. Similarly, Wiech et al. have shown that CD children adhering to GFD for a year showed a higher increase in weight and body mass index (BMI) when compared with healthy controls, suggesting a tendency towards metabolic syndrome [15]. However, there is growing evidence supporting the protective effect of GFD on bone metabolism [16] and the possible prevention of diabetes through GFD [17].

In preparing this Special Issue, GFD and fermentable oligo/di/monosaccharides and polyols (FODMAP) as dietary therapies in individuals with IBS was an issue that the Editors found to be important [18]. In a study evaluating the intake of foods containing fermentable oligo/di/monosaccharides and polyols (FODMAP) in CD patients, Roncoroni et al. confirmed that the prevalence of IBS-type symptoms among CD patients is higher than in the general population. Moreover, they demonstrated that CD patients consume a diet high on FODMAP, which is a factor that possibly induces gastrointestinal symptoms in treated CD patients [19,20]. Moreover, in the first RCT DB intervention controlled study, the same researchers showed that CD patients on GFD, but with persisting functional gastrointestinal symptoms, had a positive response to a diet low on FODMAP. Thus, GFD associated with a low-FODMAP content is beneficial, as a support therapy, for a group of CD patients with persistent gastrointestinal symptoms [21].

A number of questions still remain unanswered; namely, the modifications by GFD of the gut microbiota in different populations [22,23]; the effects of gluten intake on both gastric and gallbladder motility [24]; and the persistent motor disorders in CD patients, despite GFD, which can be explained by low-grade mucosal inflammation [25].

Several open issues regarding GFD also remain, such as, most importantly, the ingestion threshold for the amount of gluten considered tolerable has not been defined yet. Furthermore, the appropriateness of a lifelong indication to GFD, particularly for patients with sub-clinical and potential CD (i.e., not confirmed by histology), is still a matter of debate [26], especially on consideration of the impact on patients’ quality of life posed by a restrictive gluten-free diet [27]. Finally, in a study on the immunogenic potential of α-gliadins in Triticale, Ruiz-Carnicer et al. demonstrated that by substituting a natural amino acid to the most immunogenic fraction of gluten (DQ2.5-glia-a 1, DQ2.5-glia-a2, and DQ2.5-glia-a3), the toxicity of three T-cell epitopes was eliminated, while the technological properties of commercial wheat were maintained [28]. These results may offer the opportunity to generate wheat varieties with a reduced CD immunogenicity not safe for consumption by patients, but that might help to prevent the onset of CD in people that carry genetic risk factors.

In conclusion, we would like to acknowledge all the authors for their valuable contributions and the reviewers for their constructive comments. Special thanks are owed to the publishing team of *Nutrients* for their professional assistance in the development of this Special Issue.
